# To the editor: Interventional radiology in the COVID-19 era: Crisis and opportunity

**DOI:** 10.1186/s42155-020-00162-x

**Published:** 2020-09-15

**Authors:** Konstantinos Katsanos, Panagiotis Kitrou, Dimitrios Karnabatidis

**Affiliations:** Interventional Radiology, Patras University Hospital, School of Medicine, Rion, Greece

Dear editor, I read with great interest the recent publications in CVIR Endovascular from Rostampour et al. (2020) and Morgan et all. (2020), about IR in times of COVID 19. What I am, however, missing is the broader perspective of IR and COVID 19 to emphasize the position of IR as a medical specialty.

The world has been recently shaken by the global outbreak of the coronavirus 2019 pandemic. The coronavirus disease 2019 (officially known as COVID-19) is a critical and potentially fatal viral respiratory illness caused by the severe acute respiratory syndrome coronavirus 2 (SARS-CoV-2) with estimated case fatality rates of approximately 2% (Zhu et al. 2020). COVID-19 has quickly spread without discrimination across continents and nations affecting more intensively the elderly and patients with background comorbidities. In response, most governments swiftly implemented heavy-handed national lockdowns to enforce social distancing and isolation to forestall spread of the virus, whereas public healthcare systems underwent urgent restructuring and transformation to preserve vital intensive care resources and prepare for an influx of severe respiratory infections often mandating intubation and other advanced measures of organ and life support.

Consequently, outpatient clinics across all disciplines were shut and elective appointments were indefinitely deferred, while myriads of elective orthopedic, oncologic and cardiovascular surgeries were postponed for months if not years, in order to refocus centralized tertiary healthcare towards the tsunami of COVID-19 cases. Under those turbulent circumstances, most IR departments had to quickly transform themselves by abiding with revised administrative policies, implementing very stringent infection control measures, pivoting IR services towards the special needs of coronavirus infections (Morgan et al. 2020) and catering for different frequencies and often new types of interventions, like for example arterial and/or venous thromboembolic complications in the setting of COVID-19 (Bikdeli et al. 2020). Fortunately, several national IR societies published guidance to help distressed and deranged IR units cope with infection control, everyday anxiety when dealing with confirmed or suspected COVID-19 patients and the huge numbers of postponed elective cases.

The coronavirus disease emerged in the Chinese provinces of Wuhan and Hubei. Of great interest, in the Chinese language, the word “crisis” (pinyin: *wēijī*) is composed of two characters, the first one (*wēi)* representing danger and the second one (*jī)* denoting opportunity and pivot point. To paraphrase, in the midst of chaos there is also opportunity (*The Art of War,* Sun Tzu). That is clearly the case of Interventional Radiology in the current crisis of COVID-19 pandemic. Interventional Radiologists have always championed the safety and effectiveness of a vast variety of minimally invasive procedures in the vascular and non-vascular arena with undisputed and well-proven records of lower complication rates, quicker patient discharge, improved patient comfort, and ultimately higher case throughput. IR needs to capitalize on those exact benefits during the coronavirus outbreak and the ensuing aftermath across the tertiary healthcare sector.

The vast majority of IR caseload encompasses peripheral vascular, oncologic and general non-vascular workload ranging from semi-elective to more urgent cases. We would argue that the vast majority of those cases can be safely completed and discharged home as day-case procedures minimizing risk of coronavirus contagion and optimizing utilization of hospital resources (Kok et al. 2018). For example, in our department, revascularization and foot care of critical limb ischemia patients were not neglected during the lockdown by implementing swift revascularization and same day discharge in liaison with primary care physicians as necessary. A recent survey in the United Kingdom documented that availability of day-case facilities had positively influenced IR services during the pandemic lockdown with continued provision of endovascular, genitourinary and hepatobiliary services (Rostampour et al. 2020). Time- and cost-efficiency of several IR procedures (compared to competing surgical alternatives) is a major argument during negotiations with senior administrators looking to reallocate time, people and resources, and deciding on future investment or disinvestment of healthcare infrastructure.

The best estimate is that more than 28 million operations have been cancelled or postponed during the peak 12 weeks of disruption due to COVID-19, and the vast majority of those (> 90%) being treatments for benign disease. It has been argued that even if countries increased their normal surgical volume by 20% after the pandemic, it would still take a median of 45 weeks to clear the backlog of cancelled operative procedures (COVIDSurg Collaborative 2020). This is an unprecedented opportunity for several well-established, safe and expedient IR procedures in the fields of embolization for benign disease, percutaneous angioplasty, early stage tumor ablation, superficial and deep venous disease, genitourinary interventions, percutaneous spinal column treatments, and chronic pain management just to name a few examples. In addition, novel breakthrough procedures may be allowed quicker access to the clinic by reducing waiting lists of competing surgical procedures and amassing valuable clinical evidence about their safety and efficacy in shorter time periods.

For example, percutaneous arteriovenous fistula creation (also known as endoAVF) is a promising procedure completely eliminating surgical cut-down and dissection of the vessels. During the COVID-19 lockdown of operating theaters and in view of a possible second pandemic wave, most patients that were about to start hemodialysis have had their elective surgical fistula creation cancelled and are receiving outpatient insertion of a central venous catheter instead. This is worrisome and constitutes a serious setback in their standard of care as the use of central dialysis catheters is associated with increased rates of infection, catheter malfunction, central venous stenosis and occlusion, and poorer quality of dialysis care. Percutaneous fistula creation is particularly elegant as it capitalizes on additional anastomotic sites by using the deep venous system of the forearm, it avoids superficial and deep tissue scarring and may be routinely performed as a day case procedure outside the operating theater (Inston et al. 2020).

This is the chance for IR to rise and shine as one of the most innovative, cutting-edge, and efficient medical disciplines. There is a tide in the affairs of men, which, taken at the flood, leads on to fortune; omitted, all the voyage of their life is bound in shallows and in miseries (*Julius Caesar,* William Shakespeare).

## Data Availability

Not applicable.
